# Targeted Screening for Gaucher Disease in High Suspicion Patients and Clinical Profile of Screen Positives in a Large Pediatric Multispecialty Hospital

**DOI:** 10.7759/cureus.29868

**Published:** 2022-10-03

**Authors:** Suvarna Magar, Madhuri Engade, Tushar Idhate, Sachin Khambayate, Shaikh Nilofer, Ana Kalia

**Affiliations:** 1 Pediatrics, Mahatma Gandhi Missions (MGM) Medical College, MGM Institute of Health Sciences (IHS), Aurangabad, IND; 2 Pediatrics, Mahatma Gandhi Missions (MGM) Institute of Health Sciences (IHS), Aurangabad, IND; 3 Pediatric Hematology, Mahatma Gandhi Missions (MGM) Medical College, MGM Institute of Health Sciences (IHS), Aurangabad, IND; 4 Pediatrics, Amrut Bal Rugnalaya, Aurangabad, IND; 5 Pediatrics, Mahatma Gandhi Missions (MGM) Hospital, MGM Institute of Health Sciences (IHS), Aurangabad, IND

**Keywords:** inborn errors of metabolism, lysosomal storage diseases, imiglucerase, enzyme replacement therapy, gaucher disease

## Abstract

Objectives: The proposed screening study was aimed at determining the prevalence of Gaucher disease in a selected high-risk population of patients and describing the clinical profile of diagnosed patients.

Methodology: It was a prospective observational study from January 2020 to September 2022 (two years and eight months) in the genetic clinic of the pediatric department. A total of 22 patients were suspected to be having Gaucher disease based on clinical findings of hepatosplenomegaly with bicytopenia or isolated thrombocytopenia. In these patients, chronic liver disease, portal hypertension, and other hematological conditions were ruled out. Three patients with Gaucher disease applied for enzyme replacement therapy (ERT) support under India Charitable Access Program and one patient received therapy for two months. Clinical findings were compared before and after ERT. Clinical findings were noted in all patients.

Results: Among the 22 patients, nine (40.9%) patients were confirmed to be suffering from Gaucher disease with six based on enzyme assay on dry blood spot and three based on DNA mutation analysis. One patient among the screen positives received ERT for two months and was noted to have an improvement in hemoglobin and platelet count, a reduction in liver size, and better general well-being.

Conclusion: High-suspicion targeted screening of Gaucher disease in patients with splenomegaly and thrombocytopenia based on a dry blood spot enzyme assay is high yielding, effective strategy in identifying Gaucher disease patients. Clinical features were variable in severity, though a common mutation was found in the majority of patients.

## Introduction

Gaucher disease is a hereditary autosomal recessive disease due to the presence of biallelic pathogenic variants in the GBA gene coding for lysosomal enzyme acid ß-glucosidase. This leads to a congenital deficiency of the lysosomal enzyme acid ß-glucosidase, with a consequent accumulation of non-degraded glycosphingolipids (glucocerebroside) in the macrophagic cells of the reticuloendothelial system. The incidence of the disease in patients of Ashkenazi Jewish heritage varies from one in 450 to one in 1000, while in the rest of the populations, the incidence is estimated at 1:40,000 to 1:60,000 [1]. The prevalence of Gaucher disease ranges from 0.70 to 1.75 per 100,000 individuals [2]. Prevalence in India is not known. In Weinreb et al.'s study, the average life expectancy of Gaucher disease was 68 years and in splenectomized patients, it was 64 years [3].

Signs of Gaucher disease occur after an accumulation of typical macrophagic cells full of non-degraded glucocerebroside substratum, known as Gaucher’s cells, in the liver, spleen, lung, bone marrow, and bone. Gaucher disease is, however, progressive and if it is not treated, it may lead to severe morbidity due to hemorrhage and skeletal complications, liver failure, pulmonary hypertension, and sepsis, causing a reduction in quality of life and life expectancy [4].

Standard of care currently consists of enzyme replacement therapy (ERT) with the recombinant enzyme imiglucerase as it can, especially if administered during the early stages of the disease, prevent and make most symptoms, which include hepatosplenomegaly, cytopenia, bone pain, bone crisis and osteopenia, regress [5]. ERT cannot solve irreversible damages such as avascular necrosis of the bone, and liver or spleen fibrosis, underling the importance of an early diagnosis [6].

Splenomegaly and thrombocytopenia are the two most typical and frequent manifestations of Gaucher disease. The Gaucher Registry, which gathers data from more than 5000 patients worldwide suffering from Gaucher disease, shows that 86% of them present moderate or severe splenomegaly (> five times the normal volume) and 60% thrombocytopenia (<120,000/mm^3^) at diagnosis [7].

Diagnosing Gaucher disease does not require invasive tests such as bone-marrow biopsies, as the diagnosis is made by determination of acid beta-glucosidase enzyme activity on leukocytes taken from a peripheral blood sampling. Patients with Gaucher disease typically have reduced enzyme activity (<30% compared to healthy subjects). A possible problem, deriving from the fact that this test is not routinely carried out in all laboratories, has been overcome in part by the recent refining of a diagnostic method involving measuring enzyme activity in dried drops of blood on filter paper, known as dried blood spot (DBS), which has simplified the collection and transport of samples. As Gaucher disease is a rare disease, the diagnosis is often delayed as it is not suspected by clinicians. A study by Agarwal et al. from India has mentioned the delay in diagnosis and late referral as it is not suspected by clinicians [8]. So doing this project of high suspicion screening of Gaucher disease and publishing the outcomes from our genetic clinic becomes an important step for creating awareness among Indian doctors.

So we wanted to provide targeted screening for Gaucher disease in patients with unexplained splenomegaly and/or thrombocytopenia by enzyme assay on DBS in a large pediatric multispecialty hospital.

## Materials and methods

The study was approved by the Institutional Ethics Committee of our institute (EC approval number: ECRHS/2020/93). The patients were referred to the genetic clinic from various clinical specialties including pediatrics, hematology, GI, and other relevant departments. The high-suspicion cases were screened and we included patients with a clinical history of unexplained splenomegaly and/or thrombocytopenia. The screening was done by estimation of acid beta-glucosidase enzyme activity on DBS samples collected on a DBS card, which is a special filter paper provided by Sanofi Genzyme Company as a part of the project. This DBS card has eased the sample collection and transport of samples to the laboratory at a farther distance.

Inclusion criteria included patients of any age with unexplained splenomegaly, defined as clinically palpable spleen ≥ 2 cm from the costal margin, or diagnosed by ultrasound scans, MRI, or CT of the spleen, and patients with thrombocytopenia (<150,000/mm^3^).

Exclusion criteria included the cases where other known major/common causes of splenomegaly and/or thrombocytopenia have already been excluded like hematological malignancies (such as leukemia and lymphoma), splenomegaly due to portal hypertension, hemolytic anemia, and history of splenectomy for some other known cause.

All pediatric patients were referred to the genetic clinic. Once the patients were enrolled based on unexplained splenomegaly and thrombocytopenia, patients were subjected to DBS sample collection. Patients were screened for Gaucher disease by detecting the activities of glucocerebrosidase and chitotriosidase were estimated by fluorimetric assay/tandem mass spectrometry in DBS from January 2020 to August 2022. Patients who tested positive by screening test were re-confirmed through mutational analysis. All the patients were of Asian ethnicity. Patients underwent enzyme assay and mutation analysis on the DBS sample. Three patients were subjected to mutation analysis of the GBA gene by next-generation sequencing-based DNA studies without enzyme assay. Case number 4 was a neonate who initially presented as a collodion baby at birth. As collodion skin is a clinical feature of underlying ichthyosis and ichthyosis is caused by many genes, clinical exome sequencing was performed to look into all causes. Eventually, the patient developed splenomegaly, bicytopenia, and seizure disorder, which were features of neuronopathic Gaucher disease. The other two patients (case numbers 8 and 9) had anemia with splenomegaly as the predominant feature and platelet count was mildly reduced. To rule out other differential diagnoses of hemolytic anemia, we sent a clinical exome sequencing study for DNA-based diagnosis.

## Results

Among 22 patients screened for Gaucher disease, six patients were diagnosed based on DBS enzyme assay (27.2%) and three patients were subjected to mutation testing without DBS enzyme assay (13.6%). There were six males and three females. The average age of diagnosis was 15 months, with an age range from 12 days to 24 months. Six out of nine patients were born of consanguineous marriage (66.67%). The average age of presentation was 8.4 months (Table 1).

**Table 1:** Demographic and clinical characteristics of patients with Gaucher disease

Case No.	Age at diagnosis	Sex	Born of consanguineous marriage	Age at presentation
1	12 months	M	Yes	8 months
2	24 months	M	No	12 months
3	18 months	M	No	12 months
4	12 days	M	Yes	Birth
5	12 months	M	Yes	7 months
6	12 months	F	Yes	6 months
7	12 months	F	Yes	6 months
8	20 months	F	Yes	6 months
9	24 months	M	No	18 months

Table 2 shows clinical features in the patients. All patients had thrombocytopenia and mild to moderate anemia, four patients required packed cell transfusions, and one patient required platelet transfusion for thrombocytopenia. All nine patients had splenomegaly and hepatomegaly. All of them had moderate to massive splenomegaly and mild to moderate hepatomegaly, except one neonate who had mild splenomegaly and hepatomegaly.

**Table 2:** Clinical features of Gaucher’s disease

Case No.	Transfusion	Bleeding	Fracture	Hepatomegaly	Splenomegaly	Neurological	Collodion skin	Splenectomy done
1	No	No	No	Yes	Yes	No	No	No
2	Yes, packed cells and platelet transfusions	Yes, requiring platelet transfusion	Yes, spinal fracture and femur fracture	Yes, 365 cc	Yes, 1150 cc	No	No	Yes
3	No	No	No	Yes	Yes	No	No	No
4	No	No	No	Yes	Yes	Yes, multiple seizures, developmental delay	Yes	No
5	Yes, packed cells	No	No	Yes	Yes	No	No	No
6	No	No	No	Yes	Yes	No	No	No
7	Yes, packed cells	No	No	Yes	Yes	No	No	No
8	Yes, packed cells	No	No	Yes	Yes	No	No	No
9	No	No	No	Yes	Yes	No	No	No

**Figure 1:**
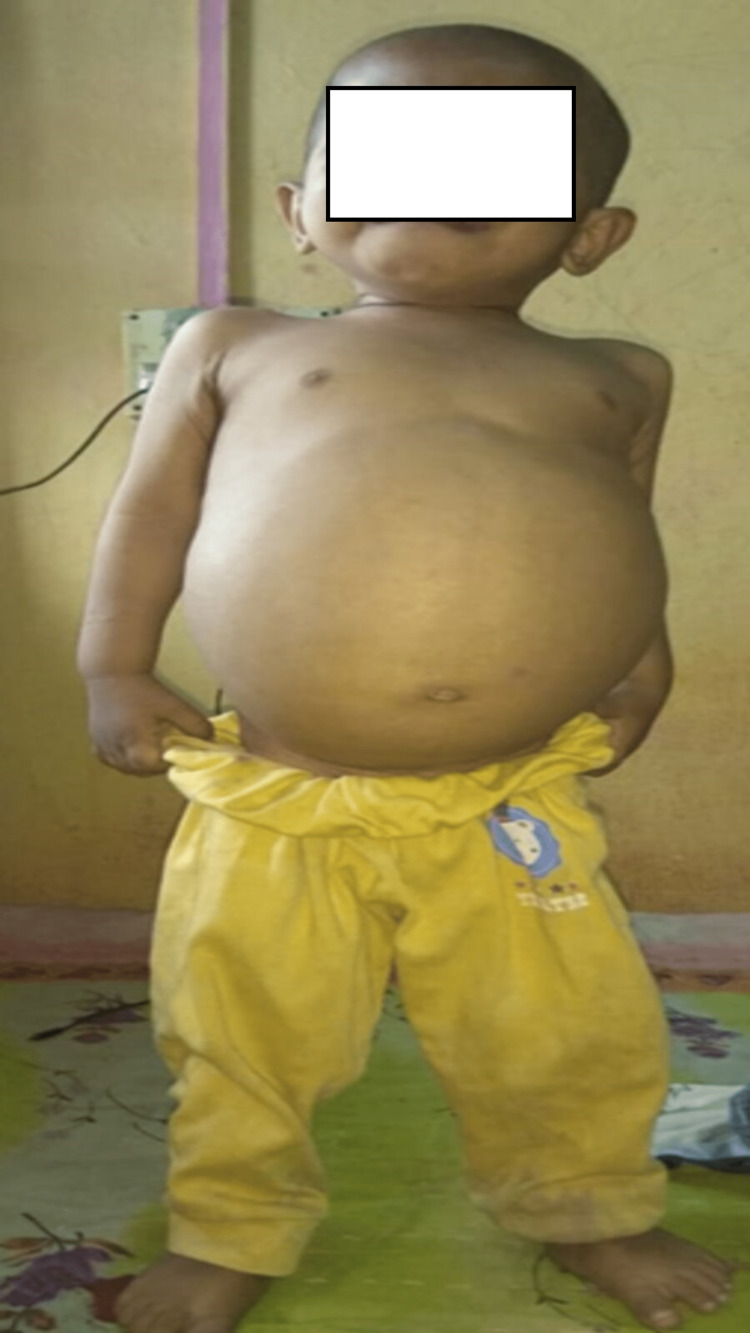
Clinical picture of case 2 who received enzyme replacement therapy

Case number 2 had massive splenomegaly with a volume of 1160 cc and moderate hepatomegaly with a volume of 365 ccs. This patient also had severely reduced bone mineral density on the dual-energy X-ray absorptiometry (DEXA) scan (z score below -3 SD). He suffered a femur fracture six months after the diagnosis and a spinal fracture two months after the ERT, which recovered after six weeks of bracing and supportive treatment. Case number 2 underwent splenectomy given massive splenomegaly with hypersplenism and bleeding tendencies with refractory thrombocytopenia and platelet count below 20,000/ccm (Figure 1).

**Figure 2:**
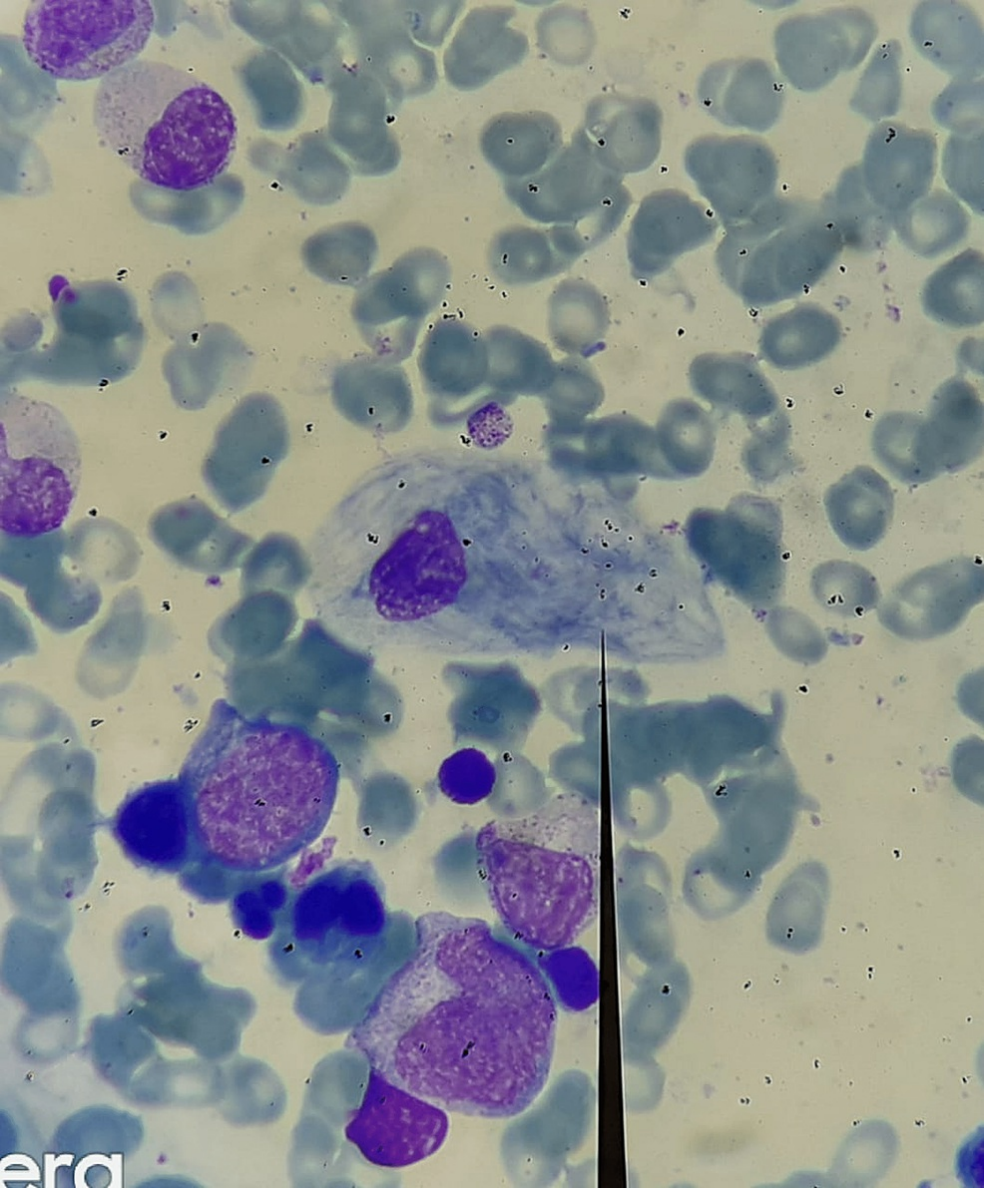
Crinkled paper macrophage or Gaucher cell in histopathology of bone marrow aspiration

Case number 4 had collodion skin at birth with hepatosplenomegaly and thrombocytopenia. This patient succumbed to encephalopathy with refractory seizures as a case of perinatally lethal Gaucher disease at the age of two months. The high hemoglobin is normal in the neonatal age and the patient did not have anemia as the patient was a neuronopathic Gaucher disease case. Anemia could have developed eventually but the patient succumbed within two months of birth. As seen in Table 3, except in case number 4, all patients had reduced levels of hemoglobin. All patients had thrombocytopenia and a few even had leukopenia with a WBC count below 4000/cmm. The DBS enzyme levels of glucocerebrosidase were severely reduced and biomarker chitotriosidase was evaluated in three cases, which was found to be elevated. A mutation study was performed by next-generation sequencing-based DNA testing in all patients. The most frequent GBA 1 gene allele was p.Leu483Pro, which was found in six out of nine (66.6%) cases (five homozygous and one heterozygous). Three patients underwent bone marrow biopsy to rule out hematological malignancy and histopathology revealed the presence of Gaucher cells or crinkled paper macrophages in the biopsy (Figure 2).

**Table 3:** Severity of Gaucher disease and mutation study Hb: hemoglobin; DBS: dried blood spot.

Case No.	Anemia (Hb < 11.5 g/dL)	Leukopenia (WBCs < 4000 per cmm)	Thrombocytopenia (platelet count < 1.5 lakhs per cmm)	Enzyme glucocerebrosidase (2.3-14.1 nmol/mL/hr)	Chitotriosidase levels (DBS, N < 200 nmol/mL/hr)	Mutation analysis, GBA gene (zygosity)
1	7.4	2510	43,000	1.68	899.0	c.1448T>C p.Leu483Pro Homozygous
2	8.3	3100	55,000	0.6	1118.0	c.254G>A p.Gly85Glu Homozygous
3	8.5	3400	130,000	0.45	19378	c.1448T>C p.Leu483Pro Homozygous
4	19.8	11,000	51,000	-	-	c.694G>T p.Gly232Trp Homozygous
5	9.3	5300	83,000	0.83	-	c.1448T>C p.Leu483Pro Homozygous
6	7.6	4300	80,000	1.73	-	c.1448T>C p.Leu483Pro Homozygous
7	4.3	4600	66,000	0.34	-	c.1448T>C p.Leu483Pro Homozygous
8	6.3	12,200	137,000	-	-	c.1504C>T (p.Arg502Cys) Homozygous
9	8.4	12,400	198,000	-	-	c.1448T>C (p.Leu483Pro) c.1483G>C (p.Ala495Pro) Compound heterozygous

The clinical manifestations and severity are described in Table 3. ERT with imiglucerase was given free of cost in case number 2 for two months under Sanofi Genzyme Company’s Charitable Access Program. The dose of imiglucerase was 60 units/kg and it was transfused once in two weeks. Response to enzyme replacement was prospectively analyzed after two months. It improved the general well-being of the child with increased activity and playfulness. Liver volume reduced to 300 ccs and complete blood count showed hemoglobin of 9 g/dL and platelet count of more than 100,000/cmm. We could not document bone scan findings because of financial constraints. ERT was uneventful requiring daycare admission with no side effects.

## Discussion

DBS, a simplified screening method to measure the glucocerebrosidase (GBA) activity using dried blood spots on filter paper, has recently been developed and studied by Miyamoto et al., Lei et al., and Motta et al. [9-11]. Collecting and sending DBS samples was very feasible in all the studies and our study too. Our genetic clinic had a majority of referrals from hematologists followed by a gastroenterologist. From the above study of high-suspicion screening of Gaucher disease enzyme assay on the DBS sample, the prevalence was 31.5%. In a similar Canadian study, no case was detected [12], a Chinese study on adults demonstrated a prevalence of 7% and a pediatric study from China demonstrated a prevalence of 23.7% [10,13]. The study by Miyamoto et al. described 76 screen positives on the first DBS, followed by 11 positives on the second DBS testing. However, they had a high cut-off value of enzyme as <3 pmol/hr/disc, which was also used in the study by Huang et al. [10,14]. Lowering the false-positive rate may not be the optimal route for detecting the incidence of a rare disease. Nevertheless, whichever cut-off is chosen, only a tentative identification of Gaucher disease can be made based on DBS testing and clinical manifestations; genetic testing is still required to confirm a definitive diagnosis. Our study had no false-positive results, as all the patients underwent genetic testing and were proven to be having Gaucher disease. Exposure of blood spots to both elevated heat and humidity can destroy enzyme functions rapidly as well as incomplete mixed blood before spotting can result in a significant variation in enzyme activities resulting in false negative results [15].

Our patient who received ERT for two months showed improved physical activity, a decrease in liver size, and an improvement in hemoglobin and platelet count. This ERT was available free of cost under India Charitable Access Program by Sanofi Genzyme Company. A study by Muranjan and Patil from India concluded that despite receiving a dose of <60 units/kg or low doses of imiglucerase within the first year of treatment and hematological reconstitution, a 10-30% reduction in liver and spleen size was seen [16]. Individuals with type 1 Gaucher disease report improved health-related quality of life after 24-48 months of ERT [12] and two months of data are inadequate to comment on the outcome. But we would like to mention here that, after a year of repeated applications to the company for the Charitable Access Program, the patient was approved for ERT for two months and was requested to apply for government funding. In India, the high cost of the ERT poses difficulties in the availability of the drug. Here, we want to highlight the burden of Gaucher disease and the lack of easy availability of ERT in India. This also highlights the need for more awareness among clinicians and more efforts by the Government of India. Our study revealed that p.Leu483Pro is the commonest mutation. Studies by Sheth et al. and Muranjan and Patil have already revealed p.Leu483Pro as the most prevalent mutation in Indian patients with type 1 Gaucher disease [16,17].

## Conclusions

High-suspicion targeted screening of Gaucher disease in patients with splenomegaly and thrombocytopenia based on a DBS enzyme assay is a high-yielding, effective strategy in identifying Gaucher disease patients. Clinical features were variable in severity, though a common mutation was found in the majority of patients.
